# The Role of Endoglin in Hepatocellular Carcinoma

**DOI:** 10.3390/ijms22063208

**Published:** 2021-03-22

**Authors:** Kuo-Shyang Jeng, I-Shyan Sheen, Shu-Sheng Lin, Chuen-Miin Leu, Chiung-Fang Chang

**Affiliations:** 1 Division of General Surgery, Far Eastern Memorial Hospital, New Taipei 22060, Taiwan; kevin.ksjeng@gmail.com (K.-S.J.); ffcc840922@gmail.com (S.-S.L.); 2 Department of Hepatogastroenterology, Chang-Gung Memorial Hospital, Linkou Medical Center, Chang-Gung University, Taoyuan city 33305, Taiwan; happy95kevin@gmail.com; 3 Institute of Microbiology and Immunology, National Yang-Ming Chiao-Tung University, Taipei city 11221, Taiwan; cmleu@ym.edu.tw

**Keywords:** endoglin, hepatocellular carcinoma, angiogenesis

## Abstract

Endoglin (CD105) is a type-1 integral transmembrane glycoprotein and coreceptor for transforming growth factor-β (TGF-β) ligands. The endoglin/TGF-β signaling pathway regulates hemostasis, cell proliferation/migration, extracellular matrix (ECM) synthesis and angiogenesis. Angiogenesis contributes to early progression, invasion, postoperative recurrence, and metastasis in hepatocellular carcinoma (HCC), one of the most widespread malignancies globally. Endoglin is overexpressed in newly formed HCC microvessels. It increases microvessel density in cirrhotic and regenerative HCC nodules. In addition, circulating endoglin is present in HCC patients, suggesting potential for use as a diagnostic or prognostic factor. HCC angiogenesis is dynamic and endoglin expression varies by stage. TRC105 (carotuximab) is an antibody against endoglin, and three of its clinical trials were related to liver diseases. A partial response was achieved when combining TRC105 with sorafenib. Although antiangiogenic therapy still carries some risks, combination therapy with endoglin inhibitors or other targeted therapies holds promise.

## 1. Introduction

Hepatocellular carcinoma (HCC) is a leading cause of global cancer mortality [1], and HCC is biochemically characterized by neo-angiogenesis, which is affected by pro-angiogenesis factors secretion by the tumor and adjacent cells [2]. Angiogenesis is the formation of new blood vessels from preexisting endothelial cells, and it is required for cancers to obtain nutrients and oxygen, and to remove waste [3,4]. Moreover, angiogenesis is a key process in tumor growth, progression, invasion, and metastasis [5].

Tumor angiogenesis is a dynamic process characterized by an imbalance of specific promoters and inhibitors. The migration, proliferation, and differentiation of endothelial cells result in neovascular sprouting [6,7], which leads to HCC tumors becoming hyper-vascularized [8]. Moreover, tumor angiogenesis contributes to early progression, invasion, postoperative recurrence, and metastasis of HCC [9]. Angiogenesis inhibition is therefore a potentially effective strategy in the treatment of HCC.

Endoglin (also known as CD105) was first identified by immunofluorescence staining of vascular endothelium [10]. The endoglin (ENG) gene is located at human chromosome 9, and the endoglin protein is a homodimer of two subunits linked with disulfide bonds, large extracellular domains, and serine/threonine-rich cytoplasmic regions [11,12]. Endoglin (CD105) is a type-1 integral transmembrane glycoprotein and coreceptor for transforming growth factor-β (TGF-β) ligands essential for fibrogenesis and angiogenesis [12,13,14,15,16]. CD105 could activate activin receptor-like kinase 1 (ALK1)/SMAD family member 1/5 (SMAD1/5) or ALK4/SMAD2/3 signaling for cell proliferation and migration, extracellular matrix (ECM) synthesis, and angiogenesis [17]. Recognized as an angiogenesis marker in endothelial cells (ECs), endoglin is also abundant in angiogenic HCC ECs. As such, this review examines recent studies on the onco-pathological role of endoglin and anti-endoglin antibodies in HCC.

## 2. Structure, Function, and Signaling Pathway of Endoglin

Endoglin, a 180 kDa homodimer, consists of a hydrophobic transmembrane domain and large extracellular domain (561 aa) containing a serine/threonine-rich cytoplasmic short tail [12,18]. There are two endoglin isoforms: long (L) and short (S). These isoforms differ in cytoplasmic domain length, cellular localization, and level of phosphorylation [18,19,20]. Short isoform endoglin (S-ENG) is induced in the senescent ECs [21]. Long isoform endoglin (L-ENG) is abundant in human liver [22], and rodent studies have shown that S-ENG acts as an anti-angiogenic agent counter to L-ENG [23]. A novel form of L-endoglin could facilitate liver fibrosis in rats [24]. Human cell line studies have shown that L-ENG activates the ALK1/SMAD1/5 signaling pathway, while S-ENG activates the ALK5/SMAD2/3 signaling pathway. It is suggested that L-ENG aggregates type I collagen and connective tissue growth factor (CTGF) in myoblasts, while S-ENG upregulates ECM synthesis via the TGF-β/ALK5 signaling pathway [25].

Transforming growth factor-β (TGF-β), a pleiotropic cytokine, is involved in cell proliferation, adhesion, cytoskeletal organization, apoptosis, and reorganization of the extracellular matrix. Given this central role in several key cellular processes, TGF-β is also recognized as playing an essential role in carcinogenesis [18,26,27,28,29]. The TGF-β superfamily consists of TGF-β serine/threonine kinase receptors (TGF-β RI and TGF-β RII), TGF-β1, TGF-β3, and some cytoplasmic proteins [18]. Endoglin interacts with the TGF-β family by binding to TGF-β RI and RII at the cytoplasmic and extracellular domains [26,30,31], as well as serving as an auxiliary receptor to form TGF-β, β-glycan, the type III TGF-β receptor (TGF-β RIII) [26,28]. In such a quaternary configuration, the receptor complex is able to modulate TGF-β, SMAD1/5/8, and SMAD2/3 signaling [32]. TGF-β and hypoxia upregulate endoglin expression, while endoglin (and β-glycan) adjusts both the inhibition and enhancement of TGF-β-related responses in certain cell types [18,33,34].

**Figure 1:**
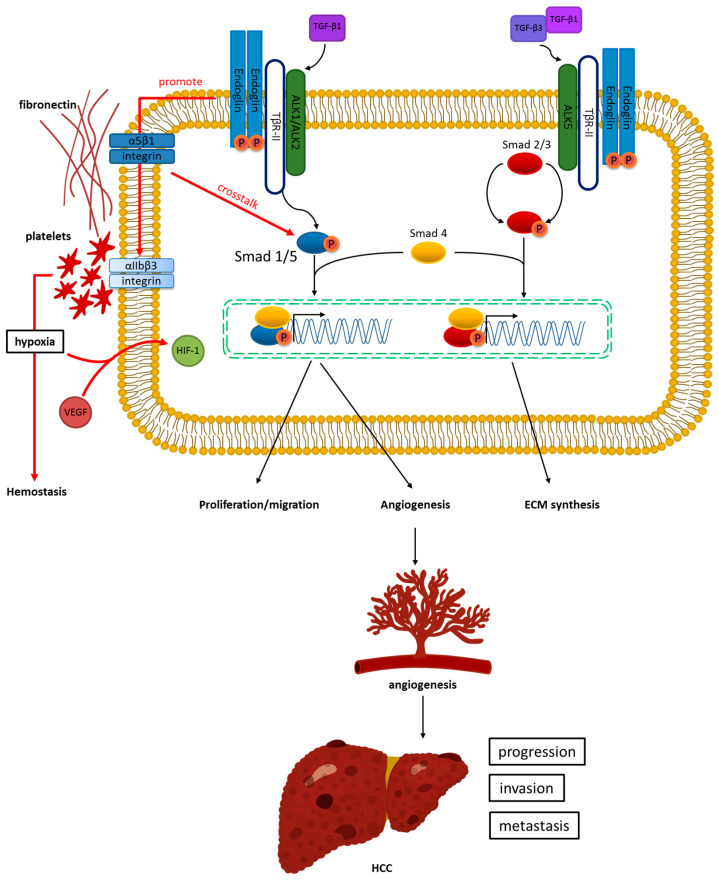
Endoglin and transforming growth factor-β (TGF-β) signaling in hepatocellular carcinoma (HCC). Endoglin binds to TGF-β1 by linking with TGF-β type II receptor activin receptor-like kinase 1 (ALK1). This leads to downstream SMAD family member 1/5 (SMAD1/5) phosphorylation with SMAD4, increasing EC proliferation, migration, and angiogenesis. This process results in HCC progression, invasion, and metastasis. In addition, endoglin-mediated fibronectin/α5β1 integrin demonstrates crosstalk with the TGF-β pathway to facilitate capillary stabilization and increase angiogenesis. Conversely, endoglin binds to TGF-β1/3 by associating with TGF-β type II receptor (ALK5). This behavior activates SMAD2/3 phosphorylation with SMAD to promote extracellular matrix (ECM) synthesis.

Biochemical and genetic EC studies have shown that endoglin is essential in the TGF-β/ALK1 signaling pathway [26]. Endoglin indirectly blocks TGF-β/ALK5 signaling via the enhancement of TGF-β/ALK1 signal transduction [26,35]. After endoglin participation in the TGF-β RI complex, the signals could be transmitted from the cellular membrane to the nucleus through phosphorylation. After translocation to the nucleus, endoglin could modulate the transcription activity of the target genes. ALK1 activation is crucial in SMAD1/5 phosphorylation to enhance the proliferation and the migration of ECs. ALK5 activates and phosphorylates SMAD2/3, and inhibits certain processes [26,28,31,35]. Without endoglin, in a quiescent nonproliferative endothelium, the TGF-β/ALK5/SMAD2/3 signaling pathway could become active, whereas, in proliferating ECs, TGF-β/ALK1/SMAD1/5/8 becomes dominant [26,35,36] (Figure 1).

In the presence of TGF-βRI and RII receptors, endoglin modulates binding among TGF-β1, TGF-β3 isoforms, β-arrestin2, bone morphogenic proteins (BMPs) -2, -7, -9, and -10, activin-A, and α5β1 integrin [18,26,27,37,38,39,40,41]. Endoglin-mediated fibronectin/α5β1 integrin crosstalks with the TGF-β pathway to facilitate capillary stabilization and increase angiogenesis [41]. Rossi et al. found that the extracellular domain of endoglin enhances specific αIIbβ3 integrin-mediated adhesion between the platelets and the endothelium, leading to vessel hemostasis and thrombotic inflammation [42]. In a mouse model, endoglin has been shown to affect nonresponsive T cells via interaction with histocompatibility complex barriers [43]. Endoglin affects EC activity independent of the TGF-β pathway by modulating cytoskeleton reorganization, inhibiting apoptosis, enhancing c-Jun N-terminal kinase 1 (JNK1) phosphorylation, and manipulating endothelial nitric oxide synthase expression [26,36]. Moreover, endoglin phosphorylation affects subcellular localization.

## 3. Angiogenesis of HCC and Its Relation to Endoglin

Normal liver parenchyma is perfused with both arterial and venous blood [16,44,45,46]. The liver sinusoids also have a dual blood supply fed by the portal vein (70%) and hepatic artery (30%) [47]. In HCC, the hepatic artery becomes the primary blood supply. The vascularization strategies of HCC include co-option, angiogenesis, vasculogenesis, and intussusceptive angiogenesis [44]. Sprouting angiogenesis is characterized as the arterialization of the tumor blood supply with capillarized sinusoids [48,49,50]. 

Arterialization (arteriogenesis) involves the development of collateral arteries overlaid with smooth muscle on pre-existing arteries [44,48]. Sinusoidal capillarization results in a distortion of the lumen and loss of endothelial cell fenestration along with increased basement membrane synthesis. These alterations cause fenestrated liver sinusoids to transform into continuous capillaries. As a consequence of this process, normal sinusoidal endothelial markers decrease [47,48,49]. The development of new vessels from tumor cells is referred to as vasculogenic mimicry (VM) [51]. Hypoxia-inducible factor 1-alpha (HIF-1 alpha) enhances VM formation in HCC via the upregulation of lysyl oxidase-like 2 (LOXL2) in a hypoxic microenvironment [52].

Endoglin (CD105) is overexpressed in newly formed HCC microvessels [53], allowing tumor angiogenesis to be quantified by observing the microvascular density (MVD) [4]. Immunohistochemistry (IHC) assays of CD34 and CD31 (pan-endothelial antibodies) have been used to evaluate areas of high vascularization, showing that CD105 overexpression correlates with neovascularization. CD34 and CD105 expression is seen in both HCC tissue and precancerous foci, while endoglin expression also increases microvessel density in cirrhotic nodules and regenerative nodules. Furthermore, microvessel density and CD34/CD105 expression differ between benign and malignant lesions [53].

Endothelial progenitor cells mobilize from surrounding vessels [54,55], and angiogenesis varies between dysplastic nodules, regenerative nodules, and mature HCC [4,49,53,56,57]. Normal liver tissue, regenerative nodules, and degenerative nodules are all fed by the portal vein, whereas HCC is mainly fed by the hepatic arteries [48]. Progression of vascularization therefore mediates cancer progression and may be regarded as a prognostic indicator [49]. Hematogenous hemostasis is primarily associated with HCC, characterized by an overgrowth of arteries and sinusoidal capillarization [8]. Liver sinusoidal ECs experience decreased hepatic sinusoidal fenestration, with capillaries becoming continuous. Collagen and laminin deposition are upregulated in endothelial cells and hepatocytes [58]. 

Endoglin participates in the neovascularization process of HCC [44,50,55,59], and is a cell proliferation marker for both vascular ECs and tumor vasculature [27,34,60,61]. Following the progression of HCC, liver sinusoid endothelial cells downregulate CD105 and CD34, resulting in a higher characteristic change of capillary endothelial cells [47,62]. As an endothelial marker, CD105 is superior to CD34 [63,64]. Some cells derived from HCC produce endoglin in vivo, resulting in the activation of endothelial cells, these cells are referred to as tumor endothelial cells [62,63,64,65].

Tumor endothelial cells have been shown to express endoglin and other angiogenesis-related genes that enhance tube formation and cellular migration [66]. With respect to HCC stage, the degree of endoglin expression is known to vary among endothelial cells [63,67,68]. Early HCC angiogenesis is characterized by hypoxia and an acidic tumor microenvironment that leads to endothelial sprouting. Later, elevated expression of HIF-1α and endoglin promoters is observed [18,33,69,70,71]. 

In well-differentiated HCC, endoglin expression was found to be the highest. Conversely, endoglin showed downregulation in poorly differentiated HCC [66]. Endoglin mainly exists in the microvessels of the tumor periphery, whereas TGF-β1 is present only within tumor hepatocytes [65]. Benetti et al. found that HCC-derived TGF-β1 functions as a chemoattractant for endothelial cells expressing endoglin and as a tumor angiogenesis promoter. It is reasonable to hypothesize that selective targeting of TGF-β1/endoglin (CD105) signaling could inhibit angiogenesis and the subsequent progression of HCC.

Microvessel density (the CD105-labeled endothelial cells) and G-protein coupled receptor 4 (GPR4) expression are significantly increased in HCC tissue compared to normal liver [8]. Colocalized expression of GPR4 and endoglin (CD105) therefore represents the presence of neovascularizing ECs. Together, these findings suggest that GPR4 and CD105 are useful HCC tumor markers and potential therapeutic targets.

Higher endoglin expression is seen in the endothelial cells of blood vessels within and peripheral to HCC [27,61]. With other solid tumor cancers such as prostate, lung (nonsmall cell), rectal, and squamous cell (oral) cancers, the endoglin-labeled microvessel density correlates with tumor progression [63,72,73,74,75]. CD105 was found with neovascularization primarily among the ECs of immature tumor vasculature, and it displayed influence over tumor angiogenesis. Thus, CD105 is a promising target for cancer therapy [27,72,76].

Compared to that of CD31 or CD34, CD105 expression in microvessels appears to be more clinically significant. There is a strong association between GPR4 expression and MVD, and sites of high GPR4 expression correspond with CD105 expression hotspots among hepatic sinusoids and endothelial cells surrounding tumor tissue (within a 2 cm margin). High expression of GPR4 is seen both within liver tumor tissue and at the tumor margins. Moreover, Yagmur et al. suggested that endoglin is a potential complementary biomarker in HCC risk assessment among those with liver cirrhosis [77].

## 4. Circulating Endoglin in HCC Patients

**Figure 2:**
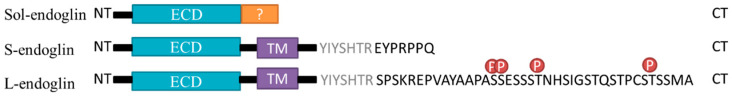
A schematic of endoglin structure. The soluble endoglin (sol-endoglin), short isoform endoglin (S-endoglin) and long isoform endoglin (L-endoglin). Relevant domains such as the extracellular domain (ECD), transmembrane domain (TM), cellular domain (CD) and phosphorylation sites by TGF-βII and ALK1 are indicated.

After proteolytic cleavage by matrix metalloproteinase 14 (MMP14), the endoglin extracellular domain takes on a soluble form (sol-ENG) (Figure 2) [78,79]. The soluble form of endoglin can then enter the bloodstream. Both endothelial cells and cancer cells release soluble endoglin [18,78]. The soluble form of endoglin can be detected in the serum of HCC patients [29,77,80,81,82,83]. Circulating serum endoglin (CD105) is also present in the cirrhotic livers of patients at risk of HCC [77]. In addition, an Egyptians study on patients with cirrhosis of the liver found that elevated endoglin and TGF-β mRNA promote hepatocarcinogenesis and increase the risk of HCC [83]. Thus, endoglin is regarded as a potential novel tumor biomarker for patients with a high risk of developing HCC.

Li et al. confirmed in both HCC tissue and the human hepatoma SMMC-7721 cell line that endoglin enhances the invasion and metastasis of HCC cells via increased expression of vascular endothelial growth factor (VEGF) [84]. The researchers also found that endoglin expression significantly correlated with tumor, node, and metastasis (TNM) staging, differentiation, portal vein invasion, and lymph node metastasis [84]. High endoglin expression in nontumor tissues suggests that such a microenvironment favors the progression of HCC. Therefore, determining tissue expression and serum concentrations of endoglin is useful for both diagnosis and prognosis.

## 5. Hepatitis C Virus Core Protein Modulates the Endoglin Signaling Pathway and the Role of Endoglin in Cancer Stem Cells, Hepatic Stellate Cells and Cancer Associated Fibroblasts

Cancer stem-like cells isolated from Hepatitis C (HCV)-infected primary hepatocytes and transformed human hepatocytes were compared, showing endoglin upregulation of up to 250-fold in sphere-forming cells (cancer stem cells) [85]. Endoglin, combined with activated phospho-SMAD1/5 and DNA binding protein 1 (ID1) was activated by the HCV core-expressing surface proteins of human hepatocytes [15]. Using an in vitro migration assay, Mardomi et al. [86] found that the combination of C-X-C Motif Chemokine 4 (CXCR4) and endoglin inhibitors mitigated the migration of bone marrow mesenchymal stem cells toward HepG2 cells. 

Kwon et al. [15] suggested that significant upregulation of endoglin (CD105) mRNA is present in HCV-associated cancer stem cells, citing an increase in endoglin expression on the cell surface of HCV core-expressing HepG2 hepatocellular carcinoma cells. Moreover, endoglin is also present on human HCV core-expressing hepatocytes, and activates phospho- SMAD1/5 and ID1 downstream signaling molecules. Upregulation of endoglin/ID1 mRNA expression was found in liver biopsy samples. Endoglin also increased cellular proliferation and migration. In HCC patients, CD105 was not only present in tumor tissues, but also abundant in hepatic sinus endothelium in nontumor tissues with cirrhosis [87]. The expression of CD105 is correlated with the expression of HIF-1α and VEGF. Recently, a novel variant with a low-frequency missense variant (Thr5Met) in the ENG gene, encoding endoglin, was associated with liver fibrosis development in HCV patients [88]. Thus, endoglin may contribute to liver fibrosis in chronic HCV-infected patients.

Endoglin expression was found to be increased in transdifferentiating hepatic stellate cells (HSCs) in a liver fibrosis model [24]. In addition, human HSC LX2 cells and hepatoma cell line HepG2 could express the full length of endoglin and direct endoglin to exosomes, requiring N-glycosylation [89]. Upon liver injury, quiescent HSCs could activate myofibroblasts. Although endoglin promotes myofibroblast differentiation in diabetic nephropathy, the role of endoglin in myofibroblasts in the liver still requires further investigation [90]. In addition, cancer associated fibroblasts (CAFs) facilitate angiogenesis in HCC [91]. Placental growth factor (PIGF) and CD90 enriched-CAFs were positively correlated with tumor angiogenesis markers (CD31, CD34, and CD105) [92]. Therefore, endoglin is a key protein in tumor growth and survival, cancer cell metastasis, and liver fibrosis.

## 6. Endoglin in Tumor-Derived Endothelial Cells and HCC

Xiong et al. [68] suggested that, compared with endothelial cells from adjacent normal tissue, tumor-derived endothelial cells are resistant to drug treatment. Compared with endoglin positive normal endothelial cells and human umbilical vein endothelial cells (HUVECs), tumor endothelial cells (TECs) expressing endoglin had a higher resistance to apoptosis, higher cell motility, and stronger proangiogenic traits. In addition, TECs expressing CD105 showed superior adherence to tumor cells and better survival in the tumor environment. Compared with endoglin-positive normal endothelial cells and human umbilical vein endothelial cells (HUVECs), TECs expressing endoglin showed greater resistance to adriamycin, 5-fluorouracil, and sorafenib treatments [68]. As such, endoglin-ALK1-SMAD1/5 axis antagonism may be an effective therapy target against liver cancer stem cells in patients with liver cirrhosis.

Endoglin CD105 microvascular density (CD105-MVD) plays a significant and independent prognostic role in those with recurrent and metastatic HCC [9]. Across the stages of carcinogenesis, endoglin was found to be overexpressed in proliferating endothelial cells of both blood and lymphatic vessels in HCC tissues. Yao et al. [63] found CD105 to be a crucial contributor to the development of HCC angiogenesis. A study by Yang et al. of HCC tissue also showed that, compared to anti-CD34, anti-CD105 (anti-endoglin) was more effective in quantifying new microvessels. 

A positive correlation between endoglin mRNA concentration and TNM clinical stage has been emphasized in the literature [9,63,83,84]. According to observations by Yang et al., there is a negative correlation between the expression of serum endoglin mRNA and the HCC clinical stage following sunitinib treatment. In patients with lower endoglin expression in serum and tissue, overall survival and progression-free survival were both significantly improved over those for cases with higher expression of endoglin. 

For HCC recurrence following liver transplantation, a higher expression of endoglin-MVD (CD105-MVD) was associated with portal vein tumor thrombus. The expression of endoglin with a diffusion pattern in the microvessels of adjacent nontumor tissue could predict early recurrence [64]. In advanced HCC cases, sunitinib treatment led to lower endoglin expression and better response to sunitinib therapy [93]. Endoglin expression in tumors and the serum endoglin (CD105) concentration may be regarded as independent predictive factors of overall and progression-free survival. A comparative study of CD105 expression found decreased expression of CD105 in tumor ECs and higher expression in nontumor ECs. Moreover, these results were prognostically significant [4,66,87,94].

Wang et al. reported higher CD105 expression in adjacent nontumor sites containing new vessels. Moreover, endoglin expression in nontumor tissue positively correlated with TNM stage, serum alpha-fetoprotein (AFP), and portal vein tumor thrombosis in HCC patients [67]. Yao et al. also found that those with HCC had a higher MVD-endoglin (CD105) score and poorer prognosis (overall and disease-free survival) than did those with a lower MVD-CD105 score [63]. Yang et al. suggested that endoglin (CD105) may therefore be used as an independent prognostic marker of survival, recurrence, and metastasis. Finally, HCC patients with lower CD105-microvessel density (CD105-MVD) had a significantly better two-year survival rate than did those with a higher CD105-MVD (47.1% vs. 13.5%) [9].

## 7. Endoglin Detection and the Inhibition of Endoglin in HCC Therapy

Zhong et al. [95] developed magnetic endoglin aptamer (mEND) imaging nanoprobes using mEND-modified magnetic carboxymethyl chitosan (CMCS) nanoparticles (mEND-Fe_3_O_4_@CMCS). The mEND-Fe_3_O_4_@CMCS nanoprobe employed a mEND to recognize the target molecule and Fe_3_O_4_@CMCS as the carrier to enable MRI-based HCC detection. These new MRI probes detecting endoglin-positive cells represent a novel technique for early HCC diagnosis.

TRC105 (carotuximab) is a chimeric immunoglobulin1 (IgG1) anti-endoglin (anti-CD105) monoclonal antibody used to inhibit angiogenesis and tumor progression by blocking endothelial cell growth. Dutty et al. combined TRC105 with sorafenib in the treatment of HCC (Table 1), finding that the two drugs could be tolerated in combination [96]. A partial response rate of 25% was achieved, suggesting that combination TRC105/sorafenib treatment is a promising strategy against HCC. 

Nanoparticles with anti-endoglin (anti-CD105) antibodies represent a new diagnostic strategy which is now often applied to HCC. Anti-CD105 antibody-gold nanoparticle targeted treatment was first reported in mice [97], and later used to study proliferation inhibition and apoptotic enhancement in hepatoma cell lines MHCC-H and HepG2. CD105-labelled docetaxel-loaded lipid microbubbles (CD105-DLLM) were shown to be effective in hepatoma cell lines following ultrasonic microbubble destruction. Western blot analysis revealed downregulated expression of proliferating cell nuclear antigen and apoptosis protein Caspase-3, as well as elevated expression of Extracellular Signal-Regulated Kinase 1/2 (ERK1/2) and p38 protein in HCC cell lines. These results suggest the activation of the mitogen-activated protein kinase (MAPK) signal transduction pathway. TRC105 was used in combination therapy with sorafenib in clinical trials [96]. ENG-target 131I anti-ENG mAb (131-I A8) pharmacokinetics are biphasic, including both a rapid distribution and gradual elimination. Treatment with 131- I anti-ENG mAb (A8) with noninvasive fluorescence imaging could serve to visualize tumors and treatment efficacy [98].

## 8. Endoglin as a Biomarker and Controversial Issues in Endoglin-Targeted Therapy

Circulating serum endoglin (CD105) could be presented in the cirrhotic livers of patients [77]. The endoglin-MVD was significantly associated with recurrent and metastatic HCC [9]. Therefore, endoglin could be a prognostic marker of angiogenesis, survival, recurrence and metastasis in HCC patients [16]. While the majority of authors favor endoglin as a target for HCC therapy, some note potential challenges. Changes in endoglin (CD105)-positive ECs occur within and around HCC, and endoglin expression can be cell-type- and HCC- stage-specific. Yang et al. emphasized that CD105 is only present in vascular HCC ECs, and is absent in normal vascular ECs [9]. Conversely, immunohistochemistry (IHC) studies by Minhajat et al. found detectability in healthy portal vessel expression (100% of patients) comparable to that of CD105 expression in cancerous sinusoidal ECs (80% of patients). Ho et al. found that large (over 5 cm) and aggressive tumors have lower MVD-CD105 scores, and typically include venous infiltration, show the presence of microsatellite nodules, and are of advanced TNM tumor stage [63,64]. Lower expression of endoglin was found in all HCC samples. There was a significantly higher expression level of endoglin (CD105) in well-differentiated HCC tissue compared with poorly and moderately differentiated HCC tissue. Conversely, some authors found an inverse correlation between MVD-CD105 and VEGF scores [94]. Qian et al. found that in patients, a negative CD105 expression level in tumor tissue indicated lower overall survival. A low CD105 level in HCC vessels may indicate severe disease, poor differentiation, or HCC progression [66]. Endoglin was present in both the neo-vessels in tumor tissues and the hepatic sinus endothelium in the nontumor tissue in those with cirrhosis. Therefore, it is suggested that endoglin is not a suitable target for anti-angiogenesis treatment in those with liver cirrhosis [87]. HCC itself is a particularly heterogenous tumor given its dual blood supply and multifactorial etiology. Moreover, anti-angiogenesis therapy also carries the risk of impacting angiogenesis elsewhere in the liver [16]. Antiangiogenic therapy therefore requires further investigation. There have been only three clinical trials in liver diseases/hepatocellular carcinoma showing partial responses. TRC105 could be tested in the right setting or in earlier stages of HCC to improve its efficacy, and the regulation of immune cell infiltration should be considered [99]. Recently, a study suggested that endoglin has an immunomodulatory role in colorectal cancer [100]. However, this remains unclear in HCC and further investigation will be required to solve this problem.

## 9. Conclusions

Endoglin plays a key role in HCC angiogenesis, cell migration, and ECM synthesis through ALK/SMAD signaling. Tumor endothelial cells express endoglin, and endoglin is upregulated in HCC stem cells. While further study remains to be done, endoglin may prove to be a useful prognostic factor for HCC. Angiogenesis is a dynamic process, and variable endoglin expression with HCC stage makes its investigation complex. Endoglin is a key protein in liver fibrosis, HCC cell growth, survival, and cancer cell metastasis. In clinical trials, TRC105 (anti-endoglin antibody) with sorafenib showed partial responses. Combination therapy with TRC105 is a promising strategy that warrants further consideration.

## Figures and Tables

**Table 1:** Clinical trials of carotuximab (TRC105) use against liver diseases.

Trial Description	Condition or Disease	FDA Approval Status	NCT#
Sorafenib and	Hepatoma	Phase 1/2	NCT01306058
TRC105 in	Liver neoplasms		
hepatocellular	Adenoma, liver		
cancer	hepatocellular carcinoma		
	Liver neoplasms, experimental		
TRC105 for	Hepatocellular carcinoma	Phase 2	NCT01375569
liver cancer	Hepatocellular cancer		
that has not responded to sorafenib	Carcinoma, hepatocellular		
Trial of TRC105 and sorafenib in patients with HCC	Hepatocellular carcinoma	Phase 1/2	NCT02560779

Treatment with NCT01306058 and NCT02560779 include sorafenib and TRC105. Treatment of NCT01375569 only includes TRC105. Condition or disease: the disease, disorder, syndrome, illness, or injury that is being studied. On ClinicalTrials.gov, conditions may also include other health-related issues, such as life span, quality of life and health risks (data from clinicaltrials.gov and TRC105 were searched using Ingenuity Pathway Analysis). NCT#: National Clinical Trial number.

## Data Availability

Not applicable.
